# Qualities or skills discriminating under 19 rugby players by playing standards: a comparative analysis of elite, sub-elite and non-rugby players using the SCRuM test battery

**DOI:** 10.1186/s13104-019-4563-y

**Published:** 2019-08-22

**Authors:** M. Chiwaridzo, G. D. Ferguson, B. C. M. Smits-Engelsman

**Affiliations:** Division of Physiotherapy, Department of Health and Rehabilitation Sciences, Faculty of Health Sciences, University of Cape Town, Cape Town, South Africa

**Keywords:** Rugby, Under 19, SCRuM, Physiological, Anthropometric, Rugby skills

## Abstract

**Objective:**

Although schoolboy rugby is growing in popularity and played at different competitive levels in Zimbabwe, the influence of playing standard on qualities or skills of older male adolescent rugby players is unknown. Utilising a cross-sectional design, this study determined anthropometric, physiological characteristics and rugby-specific game skills defining elite under 19 (U19) schoolboy rugby players. Following development and subsequent assessment of test–retest reliability of School Clinical Rugby Measure (SCRuM) test battery, this study compared performance outcomes of elite rugby players (n = 41), sub-elite rugby players (n = 46) and non-rugby athletes (n = 26) to identify qualities or skills discriminating (i) elite from sub-elite and non-rugby players, and concomitantly (ii) sub-elite from non-rugby players.

**Results:**

40 m speed test (p < 0.001, ES = 1.78) and 2 kg Medicine Ball Chest Throw test (p < 0.001, ES = 1.69) significantly discriminated elite U19 from sub-elite and non-rugby players. These tests further differentiated sub-elite from non-rugby athletes. Additionally, 1RM back squat (p = 0.009, ES = 0.57), 1RM bench press (p = 0.005, ES = 0.61), repeated high-intensity exercise test (p < 0.001, ES = 0.88) and passing ability test (p < 0.001, ES = 0.99) discriminated elite from sub-elite counterparts. These findings highlight important attributes linked to elite U19 schoolboy rugby in Zimbabwe. However, no significant differences were observed for sum of seven skinfold (p = 0.28), tackling (p = 0.08) and catching ability (p = 0.05).

## Introduction

Lately, research examining characteristics of schoolboy rugby union (RU) players has increased [1–4]. This has been necessitated by expanding participation rates in a combative sport known for high injury risk and match/training volumes [2, 5–10]. Moreover, the reported high physical and technical demands of adolescent RU [1, 5] require junior players to have optimal qualities or technical proficiencies for effective participation. Therefore, research defining key attributes important in competitive schoolboy RU is warranted especially in Zimbabwe where schoolboy RU is emerging and played at different competitive levels [11, 12]. Such evidence has implications on talent identification (TID) and long-term player development [13].

To understand player attributes important in RU, previous studies compared schoolboy RU players by playing standards at U13 [14], U16 [15] and U18 level [13, 16]. For most of these studies [14–16], the influence of playing standard was examined by comparing performance outcomes of elite adolescent RU players playing in two countries of different playing abilities. Observed differences between studies reflect differences in lifestyle, socio-economic, environmental, training philosophies, and TID initiatives among other factors. In contrast, Jones et al. [14] compared physical qualities of U18 RU players playing at different standards (academy rugby vs. school rugby) in England. The identified qualities differentiating academy from school-level RU players possibly suggest important variables contributing to a higher playing standard in U18 RU.

Given the important influence of increasing age in player characteristic development [17], U19s are an important group to target since they represent a group transitioning into senior professional rugby. To understand the attributes defining good U19 schoolboy rugby players, this study compared anthropometric and physiological characteristics across differing playing standards of elite, sub-elite and non-rugby players, and further compared rugby-specific game skills between elite and sub-elite RU players. It was hypothesised that test performances would improve significantly with increasing playing standards.

## Main text

### Study design, setting and participants

**Fig. 1:**
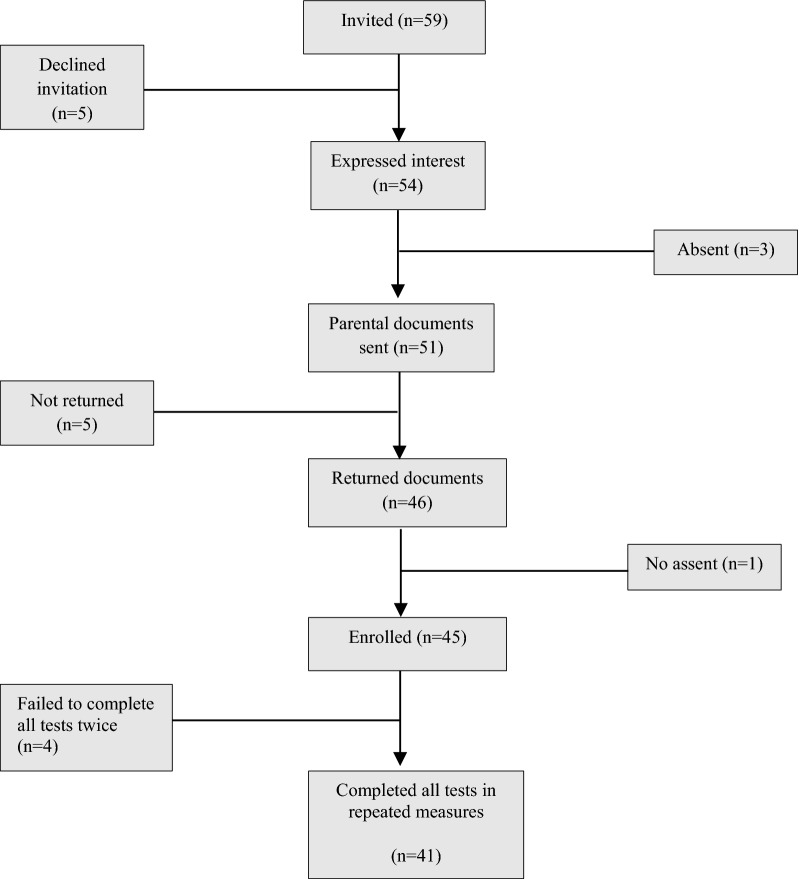
Flow chart for participants enrolled in the preliminary test–retest reliability study. Parental documents entailed Adolescent Medical Health Questionnaires and Parental Information letters

This study formed part of the School Clinical Rugby Measure (SCRuM) project described elsewhere [11, 12, 18] and was conducted in two sequential parts. Adopting a pragmatic “in-season” approach previously used by Enright et al. [19], the preliminary study established the absolute and relative reliability of each test item in the assembled SCRuM test battery. Forty-one elite U19 schoolboy rugby players completed all tests (Fig. 1) with 7 days separating test–retest assessments. The participants were recruited from one school based in Harare, Zimbabwe playing rugby in the Super Eight Schools Rugby League (SESRL). The SESRL is the most competitive schoolboy rugby league in Zimbabwe [20]. Participant testing commenced in third week from the inception of SESRL season in May 2018 (Additional file 1). Participants with self-reported injuries or any other health-related condition precluding participation in physical activity were excluded.

Utilising a cross-sectional design, the main study compared test performances of three groups of athletes. The study used baseline reliability data for elite players. Sub-elite participants were U19 male adolescent players (n = 46) recruited from a school playing in the Co-educational Schools Rugby League (CESRL). The CESRL represents a second-tier schoolboy rugby league [18]. Also, U19 schoolboy cricket players (n = 21) from one top cricket-playing school represented non-rugby athletes. The cricket players were included as a second comparative group composed of elite athletes playing a sport known to have differing physical and technical demands than rugby [21, 22]. This study was approved by University of Cape Town Human Research Ethics Committee (HREC: 016/2016). Written informed assent and consent were obtained from participants and parents respectively.

### Procedures

All eligible athletes undertook assessments in the SCRuM test battery (Additional file 2). The rationale and processes of assembling the test battery and its subsequent evaluation of face, logical validity and practical feasibility have been described elsewhere [18]. Subsequently, test–retest reliability of each SCRuM test item was established in the preliminary study using elite U19 rugby participants. Before testing, all participants were familiarised to the test battery on 2 consecutive days. Baseline results for these players were compared to data obtained from U19 sub-elite and non-rugby players. Sub-elite rugby players were tested during mid-season of the CESRL (June 2018). However, all the testing for cricket players happened during cricket competitive season (September–November 2018). The order of testing was as indicated in Additional file 1.

### Data analysis

Statistical analyses were carried out using SPSS version 25.0. Shapiro–Wilk test assessed violations of normality (p < 0.05). Descriptive statistics (Mean ± SD) described parametric data. Relative reliability was determined using two-way random intra-class correlation coefficient (ICC) for absolute agreement on single measures (Additional file 3). ICCs above 0.7 were considered acceptable [23]. Tests with low ICCs and greater coefficient of variation (CV > 10%) [24, 25] were removed. One-way analysis of variance compared group means for each playing standard. However, when equality of variance assumption was violated as assessed by Levene’s test, Welch *F* test results were reported. In case of significant effects (p < 0.05), Scheffe’s posthoc test located the mean differences with equal variances assumed. Otherwise, the Games Howell test was used. Independent *t* test compared for statistical significance between two groups. The magnitude of the differences in group means were described with Cohen’s *d* effect size (ES) calculated as the difference between group means divided by the pooled standard deviation [26]. The interpretation of ES was as follows: < 0.2 trivial, 0.2–0.6 small, > 0.6–1.2 moderate and > 1.2 large [27, 28].

### Results

Table 1 below shows group comparison for SCRuM test items.

**Table 1:** Group comparisons for demographic characteristics and SCRuM test items by playing standards

	Elite [1] (n = 41)	Sub-elite [2] (n = 46)	Non-rugby [3] (n = 21)	One-way ANOVA			[1] vs [2]. ES	[1] vs [3] ES	[2] vs. [3] ES
	Mean ± SD	Mean ± SD	Mean ± SD	F	P	Posthoc	d	d	d
Age (yrs)	17.5 ± 0.85	17.4 ± 0.87	17.6 ± 0.81	0.14	0.87		0.12	0.12	0.24
Playing exp. (yrs)	4.95 ± 0.74	4.89 ± 0.67	4.74 ± 0.38	1.56	0.24		0.09	0.36	0.28
APHV (yrs)	15.6 ± 0.60	15.8 ± 0.13	15.8 ± 0.58	1.21	0.30		0.46	0.34	0.00
Maturity offset (yrs)	1.93 ± 0.53	1.64 ± 0.97	1.78 ± 0.56	1.61	0.21		0.37	0.28	0.18
Anthropometric tests									
Body mass (kg)	77.5 ± 9.58	75.9 ± 11.6	68.5 ± 9.47	5.40	0.006*	1, 2 > 3	0.15	0.94^a^	0.70^a^
Height (m)	1.73 ± 0.06	1.72 ± 0.08	1.71 ± 0.06	0.30	0.74		0.14	0.33	0.14
Biceps (mm)	6.71 ± 3.62	6.60 ± 3.14	6.57 ± 2.27	0.02	0.98		0.03	0.05	0.01
Triceps (mm)	9.44 ± 2.95	9.83 ± 4.58	8.36 ± 2.69	1.15	0.32		0.10	0.38	0.39
Subscapul. (mm)^wg^	12.8 ± 2.74	13.5 ± 4.64	11.2 ± 2.64	3.73	0.03*	2 > 3	0.18	0.59	0.61^a^
Suprailiac (mm)	8.93 ± 3.84	9.51 ± 3.93	9.52 ± 1.98	0.34	0.71		0.15	0.19	0.00
Abdomen (mm)^w^	11.4 ± 2.85	13.3 ± 5.90	11.8 ± 2.41	1.79	0.18		0.45	0.15	0.33
Thigh (mm)	9.98 ± 2.48	11.0 ± 4.83	9.08 ± 2.00	2.86	0.07		0.27	0.40	0.52
Calf (mm)^w^	5.49 ± 1.03	6.11 ± 2.07	6.17 ± 1.29	3.08	0.05		0.38	0.58	0.03
Sum of SKF (mm)^w^	64.7 ± 15.6	69.8 ± 24.4	62.7 ± 11.6	1.30	0.28		0.25	0.15	0.37
Physiological tests									
20 m speed (s)	3.25 ± 0.17	3.36 ± 0.23^b^	3.47 ± 0.25	8.30	< 0.001*	1 < 2, 3	0.54	1.03^a^	0.46
40 m speed (s)	5.60 ± 0.29	5.84 ± 0.40^b^	6.10 ± 0.27	16.2	< 0.001*	1 < 2, 3; 2 < 3	0.69^a^	1.78^a^	0.76^a^
L-run test (s)	6.21 ± 0.32	6.33 ± 0.33^b^	6.43 ± 0.25	3.65	0.03*	1 < 3	0.37	0.77^a^	0.34
VJtest (cm)	47.8 ± 3.81	42.5 ± 3.84^b^	44.4 ± 3.85	20.5	< 0.001*	1 > 2, 3	1.39^a^	0.89^a^	0.49
2 kg MBCT test (m)	9.23 ± 1.26	8.31 ± 1.18	7.18 ± 1.16	20.4	< 0.001*	1 > 2, 3; 2 > 3	0.75^a^	1.69^a^	0.97^a^
60 s Push Up (n)	49.7 ± 9.97	43.9 ± 12.0	38.2 ± 6.50	8.95	< 0.001*	1 > 2, 3	0.53	1.37^a^	0.59
WSLS test (s)^wg^	146.0 ± 9.72	137.5 ± 21.7	132.6 ± 7.41	18.3	< 0.001*	1 > 2, 3	0.51	1.55^a^	0.30
1RM BS test (kg)	98.4 ± 14.8	89.5 ± 16.3	–	2.68^c^	0.009*		0.57	–	–
Relative BS (kg/kg^−1^)	1.27 ± 0.04	1.17 ± 0.06	–	8.77^c^	< 0.001*		1.96^a^	–	–
1RM BP test (kg)	90.5 ± 16.4	80.6 ± 15.9	–	2.86^c^	0.005*		0.61^a^	–	–
Relative BP (kg/kg^−1^)	1.16 ± 0.08	1.06 ± 0.06	–	6.98^c^	< 0.001*		1.41^a^	–	–
RHIE 1st sprint test (s)	10.2 ± 0.77	10.5 ± 0.81^b^	–	1.87^c^	0.07		0.38	–	–
RHIE 2nd sprint test (s)	13.0 ± 1.02	13.2 ± 0.96^b^	–	0.76^c^	0.45		0.20	–	–
RHIE 3rd sprint test (s)	16.1 ± 1.49	18.2 ± 1.64^b^	–	6.32^c^	< 0.001*		1.34^a^	–	–
RHIE total sprint test (s)	39.3 ± 2.96	41.9 ± 2.97^b^	–	4.04^c^	< 0.001*		0.88^a^	–	–
Decrement in RHIE (s)	5.92 ± 1.17	7.76 ± 1.31^b^	–	6.81^c^	< 0.001*		1.48^a^	–	–
Yo–Yo IRT (m)^wg^	1505.9 ± 75.8	1443.6 ± 259.1^b^	1053.3 ± 148.8	84.5	< 0.001*	1, 2 > 3	0.33	3.83^a^	1.85^a^
Rugby-specific tests									
Tackling test (%)	87.9 ± 8.44	84.8 ± 8.16	–	1.77^c^	0.08		0.37	–	–
Passing ability test (au)^wg^	116.2 ± 2.13	113.0 ± 4.07	–	4.60^c^	< 0.001*		0.99^a^	–	–
Catching ability test (au)^wg^	74.0 ± 1.07	73.5 ± 1.35	–	1.98^c^	0.05		0.41	–	–

[1], Elite group; [2], Sub-elite group; [3], Non-rugby group; Playing exp., Number of years playing competitive schoolboy sport either rugby or cricket; Subscapul. (mm), Subscapular (mm); SKF, Skinfolds; catching ability, running and catching ability test expressed in arbitrary units; SD, standard deviation; F, F test for ANOVA reporting the p value; wg, Welch F test reported and Games Howell test used for the post hoc analysis; w, Welch test results reported because of a significant Levene’s test result based on the mean; 2 kg MBCT, 2 kg medicine ball chest throw test; WSLS, Wall sit leg strength test; Yo–Yo IRT, Yo–Yo intermittent recovery test; Tackling proficiency (%), Tackling test expressed as a percentage; 1RM, one repetition maximum; 1RM BS and BP, one repetition maximum bench squat and press respectively; RHIE, repeated high intensity exercise test measured in seconds; no cricket players were allowed to perform 1RM BP, 1RM BS and RHIE because of lack of training exposure to these physically demanding tests; au, arbitrary units

^a^ Denotes moderate to large effect sizes for within age-group comparison using the Cohen d; *d,* effect size APHV, predicted age at peak height velocity based on prediction equations reflecting the estimated age at maximal velocity of growth in height during the adolescent spurt; Maturity offset (years), predicted years before or after age peak height velocity (APHV). The chronological age (CA) at prediction minus offset provides an estimate of APHV; VJ test, vertical jump test

^b^, sample size was 44 for the specified running tests

^c^, t-test independent samples test results comparing two groups; Posthoc, refers to the Scheffe or games Howell test results; Decrement in RHIE, Decrement in RHIE sprint performance calculated as the difference in time taken (seconds) to complete the third set of sprints (sprints 7–9) compared with the total time taken to complete the first set of 3 sprints (sprints 1 − 3) denoting fatigue time; d sample size was 26 for the respective tests; ES, effect size; One-way ANOVA, one way analysis of variance

* Significant p values for the ANOVA F test; 5 m speed, 10 m speed, sit and reach tests and passing for accuracy tests were found unreliable in a preliminary study involves two repeated measures. U19 cricket players did not perform game skills due to the physically and technically demanding nature of these tests and local cricket coaches’ had reservations on U19 cricket players performing rugby-oriented technical skills

### Discussion

The 40 m speed and 2 kg MBCT tests effectively discriminated elite from both sub-elite and non-rugby players, and concomitantly differentiated sub-elite from non-rugby counterparts. Additionally, 1RM BS, 1RM BP, RHIE, and passing ability skill test differentiated elite from sub-elite rugby players. Collectively, these results suggest the importance of 40 m sprinting ability, upper-body muscular power, upper-and-lower body muscular strength, repeated high-intensity performance ability and passing ability in elite U19 adolescent rugby. Practically, these findings highlight to schoolboy rugby coaches the physiological characteristics and game skills important for training for attainment of “elite” status by sub-elite or non-rugby athletes.

This study showed that elite U19s had higher absolute and relative strength compared to sub-elite players. This was despite both groups reporting equal weekly exposure to supervised resistance training. However, it is unclear whether the content or structure of the resistance training was similar or different for both groups. Moreover, there were no significant differences in playing experience and maturity between groups dismissing possible influence of biological growth and different playing experience in accounting for strength differences. Jones et al. [13] compared physical qualities of 55 U18 professional regional academy players and 129 U18 male school-level rugby players in England. Academy players recorded superior bench press values. Whether these results indicates preferable recruitment of physically stronger academy players or different strength and conditioning training practices between groups, the findings highlight the importance of upper-body muscular strength and emphasise the need for its regular training.

In the current study, elite rugby players had significantly higher 2 kg MBCT test scores compared to sub-elite and cricket players. These findings highlight the importance of muscular power development among sub-elite and potential rugby players aiming to play elite rugby. There is evidence supporting the discriminative ability of upper-body muscular power in rugby athletes of different playing abilities. For example, Till et al. [29] found significant differences in medicine ball throw distances between national and regional players in the U13 and U14 age categories. The national players representing higher-level rugby players had superior scores compared to the regional players.

Elite U19 rugby players had better 40 m speed test scores compared to sub-elite and non-rugby players. Additionally, there were meaningful ES differences between sub-elite and cricket players. These findings indicate that 40 m sprinting ability discriminates between playing levels. Hence, schoolboy rugby coaches need to implement and emphasise training strategies that maintain or maximise development of that quality especially for lower-level rugby athletes to realise elite status. However, it is unclear whether our findings suggest specialised 40 m speed training for the elite players or selection bias of players showing superior 40 m sprint abilities by schoolboy coaches in SESRL. Gabbett and Herzig [30] found contrasting results between U17 elite and sub-elite junior rugby league players. Population and sport differences could explain varied results. However, Jones et al. [13] found differences in 40 m speed test between the professional academy U18 rugby players and school-aged rugby players indicating differences in playing abilities.

Elite U19 rugby players performed significantly better on RHIE test compared to sub-elite players. These findings are expected, as the standard of rugby increases, the intensity and competitiveness increases resulting in frequent high-intensity sprinting, tackling and scrummaging episodes [31]. Gabbett [32] showed that U17 division one players engaged in more repeated high-intensity effort bouts than division three players. Depending on position, the RHIE test assesses player performance abilities on repeated sprinting, tackling and/or scrummaging facilitating understanding of physical fitness, anaerobic capacity and fatigue tolerance levels [33]. As such, elite U19 male adolescent rugby players in the present study could be highly anaerobically trained or have optimal physical fitness to tolerate match-play demands, and recover better from competition demands compared to sub-elite. Match success in rugby has been attributed to team performance on these short and repetitive high-intensity activities [34, 35]. Accordingly, the ability to intermittently engage in repeated high-intensity efforts with minimal fatigue interference should be an important attribute to train in schoolboy rugby players. However, no studies have evaluated performances of U19 schoolboy rugby players using the RHIE test. Future studies investigating the discriminative ability of RHIE test are warranted. However, it suffices to suggest for improved conditioning of sub-elite rugby athletes with regards to RHIE performance ability for the attainment of elite status in schoolboy rugby.

The present study showed that elite and sub-elite rugby players were similar in body mass and aerobic endurance but superior to non-rugby players. The lack of differences between rugby players probably suggest to the overall importance of prolonged high-intensity intermittent running ability and body mass in rugby much more than in cricket. Reportedly, rugby is a well-known high intensity, intermittent contact sport characterised by high-intensity sprints interspersed with tackles, rucks, mauls and scrums [36–38]. The present study findings align with previous studies conducted among older adolescent RU and rugby league players [13, 39–41]. Considering the physical nature of RU and the need to generate greater impact forces in collision activities, increased body size and aerobic fitness are advantageous qualities for rugby than cricket players [5, 36, 37].

Tackling proficiency and catching ability tests failed to differentiate elite from sub-elite players. However, elites had greater passing ability compared to sub-elites. This provides support for use of passing ability test for assessing playing ability in U19 schoolboy rugby. Lack of differences for tackling and catching probably emphasise the importance of these skills to the overall sport of rugby regardless of playing standard. Tackling proficiency in rugby has been related to match success [42, 43]. Consistently, Gabbett et al. [44] showed that catching and tackling skills were similar among first, second and third grade rugby league players. However, passing significantly separated first from third grade players. In contrast, Gabbett et al. [39] showed large ES differences between junior elite and sub-elite rugby players for tackling proficiency. Methodological, population and sport differences between studies could explain discordant results. In the latter study, tackling was evaluated based on technical criteria with six elements. The present study modified the criteria and had 10 items.

## Limitations

Although this study advances research on attributes defining good U19 rugby players, it is not without limitations.

i. The cross-sectional nature of the study lacked analysis over an extended period of time [45]. This design fails to consider the dynamic nature of player development possibly narrowing the usefulness of the data for TID [46].
ii. Although the novel element of this study entailed investigating SCRuM test items ability to differentiate between playing standards, one school was conveniently-selected to represent each U19 playing standard. This limits the external validity of study results to other schools and age-categories.
iii. The groups were tested at different phases of their respective seasons resulting in differences in training and competition exposure across playing standards.

## Supplementary information

**Additional file 1.** Order of the SCRuM tests performed during test–retest reliability study and subsequent studies testing rugby and cricket players.

**Additional file 2.** The SCRuM test battery.

**Additional file 3.** Results for intraclass correlation coefficient, coefficient of variation, smallest detectable change, and limits of agreement for the SCRuM test items.

## Data Availability

The datasets generated and/or analysed during the current study are not publicly available due to the fact that the data is part of ongoing research. However, the data are available from the corresponding author on reasonable request.

## Abbreviations

CESRL: co-educational School Rugby League
CV: coefficient of variation
CI: confidence interval
ES: effect size
ICC: intraclass correlation coefficient
LoA: limits of agreement
2 kg MBCT: 2 kg medicine ball chest throw
1RM BP: one repetition maximum bench press
1RM BS: one repetition maximum back squat
RHIE: repeated high intensity exercise
SEM: standard error of measurement
SESRL: Super Eight School Rugby League
SCRuM: School Clinical Rugby Measure
SD: standard deviation
SDC: smallest detectable change
SKF: skinfolds
SR: Sit and Reach test
TID: talent identification
U: under
VJ: vertical jump test
WSLS: wall sit leg strength test
Yo: Yo IRT L1-Yo–Yo intermittent recovery test level 1
