# Supplementary material for: Qualities or skills discriminating under 19 rugby players by playing standards: a comparative analysis of elite, sub-elite and non-rugby players using the SCRuM test battery
Source: BMC Res Notes. 2019 Aug 22;12:536. doi: 10.1186/s13104-019-4563-y (PMC6704687; doi:10.1186/s13104-019-4563-y)
Supplement: Supplementary file 1 — Additional file 1. Order of the SCRuM tests performed during test–retest reliability study and subsequent studies testing rugby and cricket players. [file 13104_2019_4563_MOESM1_ESM.pdf]

Order of the SCRuM tests performed during test-retest reliability study and subsequent studies testing rugby and cricket players

Group	*Week	Mon	Tues	Wedn	Thurs	Fri	Sat	Sun
E U19<br>(n=41)	Week 1	Body mass<br>Height<br>7 Skin folds<br>Sitting height	Yo-Yo<br>2kg MBCT	1RM BP<br>WSLG<br>1RM BS<br>VJ	Speed<br>SR<br>L-run	RHIE<br>Push Up	Match	Rest
	Week 2	Body mass<br>Height<br>7 Skin folds<br>Sitting height	Yo-Yo<br>2kg MBCT	1RM BP<br>WSLG<br>1RM BS<br>VJ	Speed<br>SR<br>L-run	RHIE<br>Push Up	Match	Rest
	Week 3	Tackling	Passing		Catching		Match	Rest
	Week 4	Tackling	Passing		Catching		Match	Rest
		Familiarisation of SCRuM test items to Sub-Elite U19 rugby players						
SE U19<br>(n=46)	Week 6	Body mass<br>Height<br>7 Skin folds<br>Sitting height	Yo-Yo<br>2kg MBCT	VJ<br>WSLG<br>1RM BS<br>1RM BP	Speed<br>SR<br>L-run	RHIE<br>Push Up	Match	Rest
	Week 7	Tackling	Passing		Catching		Match	Rest
	Week 8-9	***Familiarisation of SCRuM test items to Elite U16 rugby players***						
E U16<br>(n=41)	Week 10	Body mass<br>Height<br>7 Skin folds<br>Sitting height	Yo-Yo<br>2kg MBCT	VJ<br>WSLG	Speed<br>SR<br>L-run	Push Up	Match	Rest
	Week 11	Body mass<br>Height<br>7 Skin folds<br>Sitting height	Yo-Yo<br>2kg MBCT	VJ<br>WSLG	Speed<br>SR<br>L-run	Push Up	Match	Rest
	Week 12	Tackling	Passing		Catching		Match	Rest
	Week 13	Tackling	Passing		Catching		Match	Rest
	Week 14-15	***Familiarisation of SCRuM test items to sub-elite U16 rugby players***						
SE U16<br>(n=30)	Week 16	Body mass<br>Height<br>7 Skin folds<br>Sitting height	Yo-Yo<br>2kg MBCT	VJ<br>WSLG	Speed<br>SR<br>L-run	Push Up	Match	Rest
	Week 17	Tackling	Passing		Catching		Match	Rest
	Week 1-2	***Familiarisation of SCRuM test items to U19 cricket players***						
U19 Cr<br>(n=21)	Week 3	Body mass<br>Height<br>7 Skin folds<br>Sitting height	Yo-Yo<br>2kg MBCT	WSLG<br>VJ	Speed<br>SR	Push Up<br>L-run	Match	Rest
	Week 4		Passing		Catching		Match	Rest
	Week 5-6	***Familiarisation of SCRuM test items to U16 cricket players***						
U16 Cr<br>(n=29)	Week 7	Body mass<br>Height<br>7 Skin folds<br>Sitting height	Yo-Yo<br>2kg MBCT	WSLG<br>VJ	Speed<br>SR	Push Up<br>L-run	Match	Rest
	Week 8		Passing		Catching		Match	Rest

* represents the time the testing commenced which was exactly 3 weeks after the inception of the SESRL. Yo-Yo=Yo-Yo Intermittent Recovery Level 1 Test; 2kg MBCT=2kg medicine ball chest throw tests; 1RM BP=One repetition maximum bench press test; 1RM BS=One repetition maximum back squat test; WSLG=Wall Sit Leg Strength test; VJ=Vertical Jump test; SR-Sit-and-Reach test; Push Up=60s push up test; RHIE=Repeated High Intensity Exercise Performance Ability test; Match=Represents competitive match; 7 skin folds=biceps, triceps, subscapular, suprailiac, abdomen, thigh, and calf measures. Tackling=Tackling proficiency test; Passing=Passing ability and passing for accuracy for 7m test; Catching=Running and Catching Ability test. E=Elite, SE=sub-elite, Cr=Cricket; U=under;
