# Supplementary material for: Qualities or skills discriminating under 19 rugby players by playing standards: a comparative analysis of elite, sub-elite and non-rugby players using the SCRuM test battery
Source: BMC Res Notes. 2019 Aug 22;12:536. doi: 10.1186/s13104-019-4563-y (PMC6704687; doi:10.1186/s13104-019-4563-y)
Supplement: Supplementary file 3 — Additional file 3. Results for intraclass correlation coefficient, coefficient of variation, smallest detectable change, and limits of agreement for the SCRuM test items. [file 13104_2019_4563_MOESM3_ESM.pdf]

Results for intraclass correlation coefficient, coefficient of variation, smallest detectable change, and limits of agreement for the SCRuM test items

Variable	ICC	95% CI	SEM	CV (%)	$SDC_{95\%}$	LL/UL LoA
**Anthropometry**						
Body mass (kg)	1.00	0.99-1.00	0.19	0.24	0.52	-0.58/0.45
Height (m)	0.97	0.95-0.99	0.01	0.56	0.03	-0.03/0.03
Biceps (mm)	0.98	0.95-0.99	0.52	7.88	1.44	-1.20/1.68
Triceps (mm)	0.92	0.85-0.95	0.84	8.84	2.33	-2.45/2.21
Subscapular (mm)	0.96	0.92-0.98	0.55	4.29	1.53	-1.65/1.41
Suprailiac (mm)	0.98	0.96-0.99	0.54	6.05	1.51	-1.60/1.41
Abdomen (mm)	0.89	0.81-0.94	0.99	8.57	2.74	-3.01/2.47
Thigh (mm)	0.86	0.75-0.92	0.94	9.43	2.61	-2.64/2.59
Calf (mm)	0.81	0.66-0.89	0.45	8.09	1.24	-1.28/1.19
Sum of SKF (mm)	0.99	0.97-0.99	1.86	2.86	5.15	-5.59/4.71
**Physiological tests**						
5m speed (sec)	**0.52**	0.27-0.71	0.02	1.94	0.06	-0.07/0.05
10m speed (sec)	**0.64**	0.42-0.79	0.09	4.50	0.25	-0.28/0.23
20m speed (sec)	0.90	0.81-0.94	0.06	1.83	0.16	-0.13/0.19
40m speed (sec)	0.97	0.94-0.98	0.05	0.95	0.15	-0.14/0.18
L-run agility (sec)	0.90	0.82-0.95	0.11	1.72	0.30	-0.28/0.30
Vertical jump (cm)	0.93	0.88-0.96	0.97	2.03	2.70	-3.02/2.38
Sit-and-Reach (cm)	0.91	0.84-0.95	1.42	17.3	3.93	-4.57/3.30
2kg MBCT (m)	0.89	0.80-0.94	0.42	4.48	1.16	-1.34/0.98
60s Push-Up (sec)	0.93	0.88-0.96	2.59	5.15	7.17	-8.19/6.15
WSLS (sec)	0.88	0.76-0.94	2.90	1.98	8.05	-9.91/6.20
1RM BS (kg)	0.98	0.96-0.99	2.08	2.11	5.77	-6.20/5.32
1RM BP (kg)	0.98	0.96-0.99	2.40	2.64	6.64	-6.99/6.29
RHIE (sec)	0.79	0.65-0.89	1.28	3.24	3.55	-3.92/3.18
Yo-Yo IRT (m)	0.72	0.53-0.84	42.88	2.83	118.87	-135.5/102.3
**Game skills tests**						
*Tackling (%)	0.86	0.74-0.93	0.84	4.75	2.34	-2.65/2.01
Passing Ability (au)	0.71	0.52-0.83	0.98	0.84	2.71	-2.99/2.41
Pass Accuracy (%)	**0.49**	0.22-0.69	4.66	5.17	12.91	-14.36/11.44
Catching Ability (au)	0.70	0.37-0.81	0.54	0.72	1.49	-1.71/1.27

ICC=intraclass correlation coefficient; 95% CI=95% confidence interval; SEM=standard error of measurement. The SEM provided expected trial to trial measurement error and was computed as standard deviation of the differences (SDdifferences) divided √2. CV=coefficient of variation; SDC=smallest detectable change. The SDC95% for each test item was calculated by multiplying SEM with 1.96 and √2. The usefulness of the each SCRuM test item was judged by comparing the SDC95% with the SEM; The coefficient of variation expressed the SEM as a percentage of the grand mean; LoA=level of agreement; *ICC value expresses absolute agreement for average measures; BMI=Body mass index, The 95% limits of agreement (LoA) for each test item were calculated as the mean bias ± (1.96*SDdifferences); LL/UL LoA= 95% Lower limit/Upper limit Limits of Agreement, LL was calculated as mean bias-1.96*standard deviation differences; UL was calculated as mean bias+1.96*standard deviation differences; bold values represents low ICCs.
